# Fluoxetine exposure throughout neurodevelopment differentially influences basilar dendritic morphology in the motor and prefrontal cortices

**DOI:** 10.1038/s41598-022-11614-w

**Published:** 2022-05-09

**Authors:** Susan E. Maloney, Dora R. Tabachnick, Christine Jakes, Selma Avdagic, Amy L. Bauernfeind, Joseph D. Dougherty

**Affiliations:** 1 Department of Psychiatry, Washington University School of Medicine, 660 S. Euclid Ave., Campus Box 8232, St. Louis, MO 63110-1093 USA; 2 Intellectual and Developmental Disorders Research Center, Washington University School of Medicine, St. Louis, MO 63110 USA; 3 Department of Genetics, Washington University School of Medicine, St. Louis, MO 63110 USA; 4 Department of Neuroscience, Washington University School of Medicine, St. Louis, MO 63110 USA; 5 Department of Anthropology, Washington University in St. Louis, St. Louis, MO 63110 USA

**Keywords:** Brain, Cellular neuroscience, Neuronal development

## Abstract

The significance of serotonin (5HT) in mental health is underscored by the serotonergic action of many classes of psychiatric medication. 5HT is known to have a significant role in neurodevelopment, thus 5HT disruption during development may have a long term impact on brain structure and circuits. We previously generated a model of 5HT alteration throughout neurodevelopment by maternal administration of the selective serotonin reuptake inhibitor fluoxetine. We found resulting social behavior alterations in the offspring during both postnatal and adult ages. Previous work by others has indicated that early 5HT disruption influences neuronal morphology. Therefore, in the current study we sought to determine if dendritic morphological changes occur in areas involved in the social behavior deficits we previously observed, specifically the primary motor (M1) and medial prefrontal (mPFC) cortices. We quantified dendritic morphology of projection neurons in M1 and mPFC at postnatal day (P)10 and P79 in mice exposed to fluoxetine. Basilar dendritic complexity and spine density were persistently decreased in M1 fluoxetine-exposed neurons while in the mPFC, similar reductions were observed at P79 but were not present at P10. Our findings underscore that the developing brain, specifically the projection cortex, is vulnerable to 5HT system perturbation, which may be related to later behavioral disruptions.

## Introduction

Serotonin (5-hydroxytrypamine; 5HT) is one of the most well-studied neurotransmitters influencing mental health^1^, and it is a critical target for treatment of psychiatric symptoms^2–4^. During prenatal neurodevelopment, 5HT is one of the earliest neuromodulators to become active, and 5HT levels and the expression of the 5HT transporter and 5HT receptors are at their peak, allowing 5HT to modulate critical neurodevelopmental processes such as neurogenesis, apoptosis, dendritic refinement, cell migration, and synaptic plasticity^5,6^, and ultimately the construction of important behavioral circuits. 5HT also regulates the development of its own system through a negative feedback mechanism^7^. During this time, the placenta is a transient source of 5HT for the fetal forebrain until it is innervated by 5HT-producing raphe fibers^8^. In addition, increased 5HT transfer from the placenta has been shown to alter raphe innervation of the forebrain^9^. The neurons of the mature 5HT system innervate the entire CNS, influencing a variety of functions like social reward, sleep–wake cycle, perception, appetite, aggression, sexual behavior, sensorimotor activity, pain sensitivity, mood, and learning and memory^10–12^. Thus, alterations to either exogenous maternal or endogenous fetal sources of 5HT can impact the development of a range of behavioral circuits, possibly increasing risk for psychiatric outcomes.

Social behavior responses are disrupted in nearly all psychiatric disorders, particularly neurodevelopmental disorders (NDDs)^13–16^. 5HT helps to direct behavioral responses to environmental stimuli, likely through its influence on neuroplasticity^17^. Elevated 5HT activity, such as that induced by selective serotonin reuptake inhibitor (SSRI) use during pregnancy, may disrupt required sensitivity needed for proper behavior circuit development. Indeed, we previously reported altered cortical responses to somatosensory stimulation^18^, as well as early and late social behavioral deficits in a mouse model of maternal SSRI exposure during pregnancy and lactation^19^. In our model, mouse dams were exposed to fluoxetine (FLX), a common SSRI, through gestation and into the neonatal period. Thus, the developing brains of the offspring were exposed to a 5HT-activing compound through placental transfer and then lactation. In the offspring, FLX exposure reduced early social communication displays of ultrasonic vocalizations (USVs) during early postnatal life and disrupted social hierarchy behaviors in adulthood^19^.

Here, we used this model to do a next phase interrogation of the role of 5HT in the perturbation of developmental neuronal architecture, focusing on the cortical regions associated with the specific social behavior deficits we previously observed. We examined the basilar dendritic morphology of projection neurons in brain areas previously shown to be involved in USV and social hierarchy behaviors. Specifically, we found morphological changes in the basilar dendrites of layer V pyramidal neurons of the primary motor cortex (M1) at the second week postnatal that persisted into adulthood, as well as in neurons of the medial prefrontal cortex (mPFC) in adulthood. Our findings suggest that FLX’s influence on basilar dendritic morphology may underlie the behavioral changes we previously uncovered. Understanding the morphological changes that occur in the brain due to SSRI exposure could aid our understanding of when the 5HT system is most vulnerable to perturbation and the developing brain most at risk for later significant behavioral disruptions.

## Results

**Figure 1:**
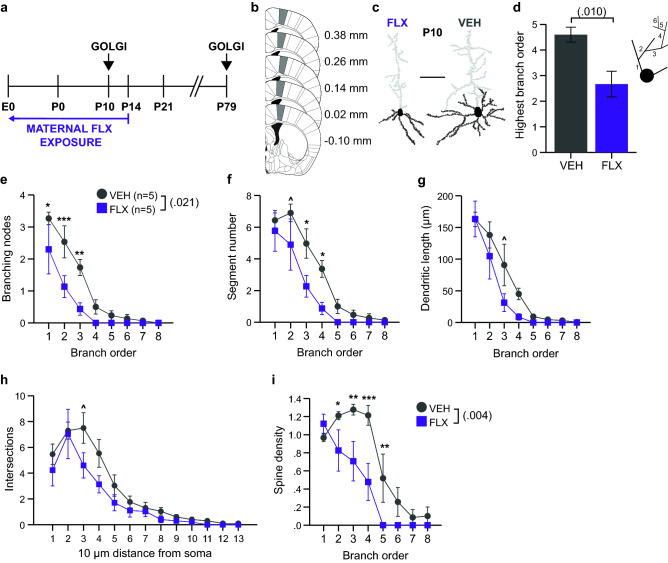
Maternal FLX reduces the extent of basilar dendritic branching and spine density of M1 layer V pyramidal neurons at P10. (**a**) Schematic of the experimental paradigm. (**b**) M1 area used for neuron selection marked in grey; adapted from Franklin and Paxinos, 2008^48^. Numbers indicate distance from bregma. (**c**) Representative tracings of Golgi-Cox impregnated M1 layer V neurons from P10 FLX and VEH brains. Areas in black indicate quantified basilar dendrites and soma. Scale bars are 50 µm. (**d**) FLX neurons did not exhibit the extent of basilar dendritic branching as VEH control neurons at P10 (n = 10 neurons averaged from 5 FLX mice and n = 10 neurons averaged from 5 VEH mice). Inset: schematic of dendritic branch orders, where 1st order branches initiate at the soma, and each subsequent branch order extends from the previous, lower order. (**e**) Fewer basilar branching nodes were observed in FLX-exposed neurons compared to VEH-exposed neurons, with particularly robust deficits at 1st, 2nd, and 3rd branch orders. (**f**) Number of dendritic segments per branch order were reduced in FLX neurons at orders 2, 3 and 4. (**g**) Comparable basilar dendritic length per branch order was observed between FLX- and VEH-exposed neurons. (**h**) Number of intersections with concentric circles per 10 µm distance from the soma as assessed by Sholl analysis did not differ between drug groups. (**i**) FLX-exposed neurons showed significantly reduced spine density over all with the greatest differences occurring at orders 2–5. Data are means ± SEM. Statistical significance, ***p < .001, **p < .01, *p < .05.

We examined the neuronal morphologies of mice exposed to FLX during brain development through dam drinking water to identify long-term cellular changes within key cortical regions (Fig. 1a). We hypothesized that dendritic morphology would be altered in areas of the mouse brain relevant to the social behavior deficits that we observed in our previous work^19^. The apical dendrite tracings were often truncated; therefore, only morphological features of the basilar dendrites were quantified.

### Maternal FLX results in comparable pregnancy to VEH exposure

To understand if exposure to the drug differentially influenced the pregnancies of the dams, we analyzed various outcomes related to pregnancy and drug exposure. We did not find any differences between the VEH- and FLX-exposed dams in this study in breeding weight, average daily consumption of drug/vehicle water, number of live births, or gestational duration (see Table 1). Thus, we conclude that FLX exposure resulted in comparable pregnancies to VEH exposure.

**Table 1:** Pregnancy outcomes between FLX- and VEH-exposed dams.

Pregnancy Outcome	VEH (*n* = 4)		FLX (*n* = 3)		Mann–Whitney U Test		
	*M*	*SD*	*M*	*SD*	*U* statistic	*z* score	*p* value
Dam Breeding Weight (g)	24.36	2.48	20.63	3.99	3	−1.061	0.400
Avg. Daily Drug Water Consumed (g)	7.77	1.58	6.81	0.66	4	−0.707	0.629
Pups in Litter	5.75	0.96	8.00	1.73	11	1.852	0.114
Gestation Duration (day)	21.00	1.15	21.67	1.53	8	0.764	0.629

### Morphological changes observed in M1 layer V neuron dendrites in the early postnatal brain following maternal FLX persist into adulthood

To follow up on our previous observation of a robust decrease in USVs at postnatal day (P)5-P9 in mice exposed to maternal FLX, we examined whether there were corresponding morphological changes in layer V pyramidal neurons in M1 at a similar age (Fig. 1b). We chose this area based on previous work that sought to identify the circuitry underlying USVs. The specific portion of M1 sampled here corresponded to the area which sends projections to the nucleus ambiguus, the motor nucleus of the hindbrain that innervates the muscles of the soft palate, pharynx, and larynx, which is active during adult mouse USVs^20^. We quantified the dendritic morphology of these neurons at P10 (Fig. 1c; Tables 2 and 3). The size of the neuron soma was comparable for FLX- and vehicle (VEH)-exposed neurons (Table 3). The extent of basilar dendrite branching in neurons from FLX-exposed brains was reduced. Specifically, there were fewer higher order branches in the FLX-exposed neurons as compared to the VEH-exposed neurons (Fig. 1d). In parallel, fewer total branching nodes were observed, particularly those that create 2nd, 3rd, and 4th order branching (Fig. 1e), which was reflected fewer 2nd, 3rd, and 4th order branch segments (Fig. 1f). The differences in overall length of basilar dendrites and dendritic length across branch orders in P10 M1 neurons were not significant (Table 2; Fig. 1g). Application of the Sholl method to spatially analyze complexity revealed comparable dendritic intersections with the concentric circles at 10 µm intervals (Fig. 1h). Finally, reduced spine density was observed across dendritic branch orders of FLX neurons, with robust differences at 2nd, 3rd, 4th and 5th orders (Fig. 1i).

**Table 2:** Statistical analysis results from all experiments.

Figure	Variable	Comparison	Statistical Test	Output	*p* value
Table 3	P10 M1 soma area	Drug	ANOVA	*F*(1,8) = .384	0.553
1	P10 M1 highest branch order	Drug	ANOVA	*F*(1,8) = 11.066	0.010
	P10 M1 branching nodes	Drug	rmANOVA	*F*(1,8) = 8.210	0.021
		Drug x Order		*F*(7,56) = 2.610	0.021
		1st Order, Drug	Simple main effect	*F*(1,64) = 6.279	0.015
		2nd Order, Drug	Simple main effect	*F*(1,64) = 13.170	0.0006
		3rd Order, Drug	Simple main effect	*F*(1,64) = 11.356	0.0013
	P10 M1 branch segments	Drug	rmANOVA	*F*(1,8) = 4.601	0.064
		2nd Order, Drug	Simple main effect	*F*(1,64) = 4.063	0.288^‡^
		3rd Order, Drug	Simple main effect	*F*(1,64) = 8.389	0.040^‡^
		4th Order, Drug	Simple main effect	*F*(1,64) = 7.193	0.072^‡^
	P10 M1 branch length	Drug	rmANOVA	*F*(1,8) = 2.570	0.148
		3rd Order, Drug	Simple main effect	*F*(1,64) = 6.970	0.080^‡^
	P10 M1 Intersections	Drug	rmANOVA	*F*(1,8) = 2.156	0.180
		30 µm, Drug	Simple main effect	*F*(1,104) = 8.822	0.052^‡^
	P10 M1 spine density	Drug	rmANOVA	*F*(1,8) = 15.502	0.004
		Drug x Order		*F*(5.5,44.2) = 2.597	0.034
		2nd Order, Drug	Simple main effect	*F*(1,64) = 4.135	0.046
		3rd Order, Drug	Simple main effect	*F*(164) = 9.089	0.004
		4th Order, Drug	Simple main effect	*F*(1,64) = 15.182	0.0002
		5th Order, Drug	Simple main effect	*F*(1,64) = 7.540	0.008
Table 3	P79 M1 soma area	Drug	ANOVA	*F*(1,7) = .032	0.863
2	P79 M1 highest branch order	Drug	ANOVA	*F*(1,7) = .018	0.898
	P79 M1 branching nodes	Drug	rmANOVA	*F*(1,7) = 4.666	0.068
	P79 M1 branch segments	Drug	rmANOVA	*F*(1,7) = 4.201	0.080
	P79 M1 branch length	Drug	rmANOVA	*F*(1,7) = 1.605	0.246
	P79 M1 Intersections	Drug	rmANOVA	*F*(1,7) = 2.264	0.176
	P79 M1 spine density	Drug	rmANOVA	*F*(1,7) = .065	0.806
	P79 M1 spine numbers	Drug	rmANOVA	*F*(1,7) = 6.032	0.044
Table3	P79 mPFC soma area	Drug	ANOVA	*F*(1,8) = 3.458	0.100
3	P79 mPFC highest branch order	Drug	ANOVA	*F*(1,8) = 14.227	0.005
	P79 mPFC branching nodes	Drug	rmANOVA	*F*(1,8) = 4.256	0.073
		1st Order, Drug	Simple main effect	*F*(1,48) = 6.312	0.090^‡^
	P79 mPFC branch segments	Drug	rmANOVA	*F*(1,8) = 4.997	0.056
		2nd Order, Drug	Simple main effect	*F*(1,48) = 5.072	0.174^‡^
	P79 mPFC branch length	Drug	rmANOVA	*F*(1,8) = 1.004	0.346
	P79 mPFC Intersections	Drug	rmANOVA	*F*(1,8) = .225	0.648
	P79 mPFC spine density	Drug	rmANOVA	*F*(1,8) = 5.264	0.053
Table 3	P10 mPFC soma area	Drug	ANOVA	*F*(1,8) = .2.708	0.138
4	P10 mPFC highest branch order	Drug	ANOVA	*F*(1,8) = .200	0.760
	P10 M1 branching nodes	Drug	rmANOVA	*F*(1,8) = .241	0.637
		Drug x Order		*F*(8,64) = 2.362	0.027
		1st Order, Drug	Simple main effect	*F*(1,72) = 16.187	0.0001
	P10 mPFC branch segments	Drug	rmANOVA	*F*(1,8) = .658	0.441
		Drug x Order		*F*(8,64) = 2.145	0.044
		1st Order, Drug	Simple main effect	*F*(1,72) = 4.509	0.037
		2nd Order, Drug	Simple main effect	*F*(1,72) = 10.601	0.002
	P10 mPFC Intersections	Drug	rmANOVA	*F*(1,8) = .679	0.434
		Drug x Distance		*F*(19,152) = 1.968	0.013
		20 µm, Drug	Simple main effect	*F*(1,160) = 16.446	0.00008
		30 µm, Drug	Simple main effect	*F*(1,160) = 6.520	0.012
		40 µm, Drug	Simple main effect	*F*(1,160) = 5.537	0.020
	P10 mPFC branch length	Drug	rmANOVA	*F*(1,8) = 1.102	0.324
	P10 mPFC spine numbers	Drug	rmANOVA	*F*(1,8) = .220	0.652

^‡^Indicates Bonferroni corrected observed p value.

**Table 3:** Morphometric feature summary of layer V pyramidal neurons.

Basilar dendritic parameter		P10 VEH	P10 FLX	P79 VEH	P79 FLX
		(n = 5 M1, n = 5 mPFC)	(n = 5 M1, n = 5 mPFC)	(n = 4 M1, n = 5 mPFC)	(n = 5 M1, n = 5 mPFC)
M1	Soma area (µm^2^)	227.27 ± 19.77	207.47 ± 25.09	214.21 ± 14	219.15 ± 21.85
	Nodes	8.47 ± 1.06*	3.87 ± 1.21*	7.58 ± 1.25^	5 ± 0.42^
	Endings	15.07 ± 1.47	9.93 ± 2.58	16.71 ± 2.35	12.5 ± 1.24
	Length (µm)	454.16 ± 51.13	308.12 ± 75.4	871.37 ± 123.36	668.18 ± 104.31
	Spines	542.87 ± 73.62^	338.43 ± 77.59^	1246.25 ± 92.88*	755.53 ± 160.85*
	Spine density	1.12 ± 0.04	1.18 ± 0.08	1.52 ± 0.08	1.28 ± 0.29
mPFC	Soma area (µm^2^)	265.44 ± 13.37	345.4 ± 46.72	337.51 ± 24.72	291.14 ± 3.28
	Nodes	3.7 ± 1.37	4.47 ± 0.76	9.8 ± 1.11^	6.83 ± 0.91^
	Endings	10.6 ± 2.16	13.27 ± 1.81	18.95 ± 1.85^	14.87 ± 0.59^
	Length (µm)	264.19 ± 58.2	350.13 ± 57.57	925.71 ± 116.19	750.16 ± 131.17
	Spines	259.7 ± 77.91	419.93 ± 91.21	1322.45 ± 160.1	1016.6 ± 189.23
	Spine density	0.94 ± 0.08^	1.15 ± 0.07^	1.47 ± 0.12	1.37 ± 0.09

Results are mean ± SEM. *p < .05, ^*p* < .10.

**Figure 2:**
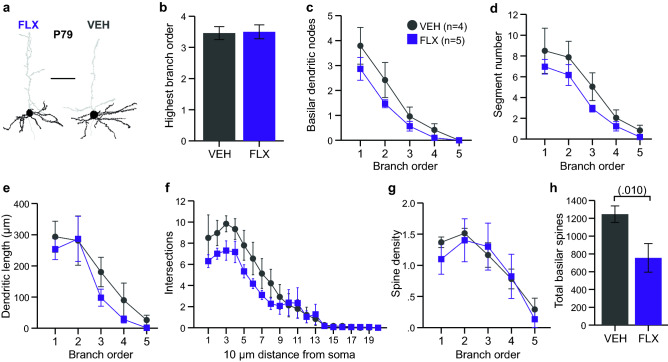
Maternal FLX impact on M1 pyramidal neurons is persistently by decreased by P79. (**a**) Representative tracings of Golgi-Cox impregnated mPFC layer V neurons from P79 FLX and VEH brains. Areas in black indicate quantified basilar dendrites and soma. Scale bar is 50 µm. (**b**) A comparable extent of basilar dendritic branching was observed in P79 VEH- and FLX-exposed neurons of the M1 (n = 10 neurons averaged from 4 VEH mice and n = 10 neurons averaged from 5 FLX mice). (**c-d**) Number of basilar branching nodes and dendritic segments per basilar dendritic branch order were non-significantly reduced in FLX-exposed neurons compared to VEH-exposed neurons at P79. (**e–f**) Basilar dendritic length per branch order and number of intersections per 10 µm distance from the soma were unchanged in FLX-exposed neurons at P79. (**g**) FLX-exposure did not influence spine density per basilar dendritic branch order in P79 neurons. (**h**) Total number of basilar spines was reduced in FLX-exposed neurons compared to VEH-exposed neurons at P79. Data are means ± SEM.

We also quantified dendritic features of M1 layer V projection neuron in adult P79 mouse brains (Fig. 1b, 2a) to determine if alterations to morphology are transient or persistent. Again, we did not observe differences in soma size between drug groups (Table 3). We also failed to observe a change to the extent of basilar dendritic branching (Fig. 2b). However, a non-significant reduction in branching nodes and branch segments were observed in FLX-exposed neurons (Fig. 2c,d). Similar to that observed in P10 M1 neurons, no differences were observed for branch length or dendritic intersections (Fig. 2e,f). In adult M1 neurons, we did not observe a reduction in spine density similar to that observed in P10 neurons (Fig. 2g), yet a significant reduction in overall number of spines was observed (Fig. 2h).

Taken together, our data on M1 layer V neurons indicate FLX exposure during brain development reduced complexity of basilar dendritic branching and impacted spine numbers that appears to have at least partially persisted into adulthood.

### Maternal FLX results in decreased dendritic branching complexity and spine density in adult mice in mPFC layer V pyramidal neurons

**Figure 3:**
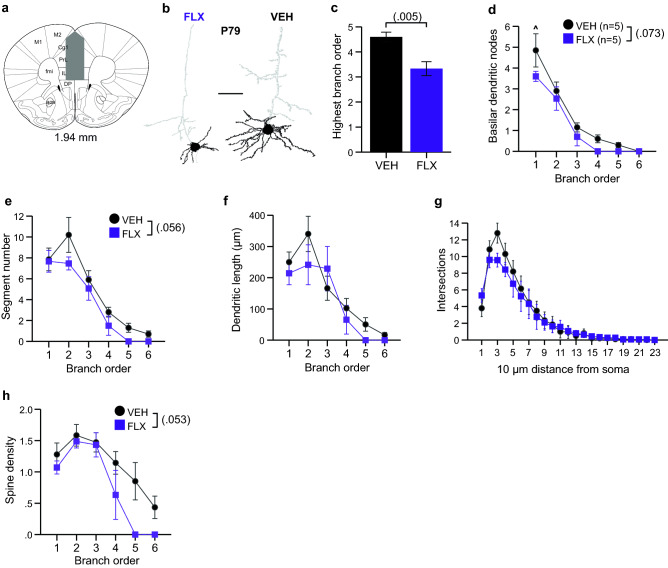
Maternal FLX results in decreased dendritic branching complexity and spine density in adult mice in mPFC layer V pyramidal neurons. (**a**) mPFC area used for neuron selection marked in grey; adapted from Franklin and Paxinos, 2008^48^. Number indicates distance from bregma. (**b**) Representative tracings of Golgi-Cox impregnated mPFC layer V neurons from P79 FLX and VEH brains. Areas in black indicate quantified basilar dendrites and soma. Scale bar is 50 µm. (**c**) P79 FLX-exposed layer V neurons failed to reach the same extent of basilar dendritic branching as VEH-exposed neurons. (n = 10 neurons averaged from 5 VEH mice and n = 10 neurons averaged from 5 FLX mice). (**d-e**) Number of basilar branching nodes and dendritic segments per basilar dendritic branch order were non-significantly reduced in FLX-exposed neurons compared to VEH-exposed neurons at P79. (**f-g**) FLX exposure failed to impact basilar dendritic length per branch order or number of intersections per 10 µm distance from the soma in P79 mPFC neurons. (**h**) Spine density per basilar dendritic branch order was non-significantly reduced at P79. Data are means ± SEM.

In our previous work, we found that our model of maternal FLX exposure affected social hierarchy norms in adulthood, specifically resulting in an increased display of social dominance compared to controls^19^. Previous research also showed that excitatory synaptic efficacy of layer V pyramidal cells in the mPFC regulated social dominance rank in the mouse^21^. Therefore, to determine if perinatal FLX exposure altered features of these neurons at a similar age at which social dominance alterations were previously observed, we quantified their dendritic morphology at P79 (Fig. 3a,b; Tables 2 and 3). The soma size of FLX neurons was smaller compared to VEH-exposed, although this difference did not reach statistical significance (Table 3). We also observed a reduction in the extent of overall basilar dendritic branching in that the highest branch order of FLX dendrites failed to reach that of VEH dendrites (Fig. 3c). In parallel, a non-significant reduction in overall nodes was observed (Fig. 3d), as well as in branch segment numbers (Fig. 3e), which was particularly robust for 2nd order segments. The overall length of basilar dendrites and dendritic length across branch orders were unchanged in FLX-exposed neurons (Table 3; Fig. 3f). Comparable intersections were observed with Sholl analysis, suggesting that spatially these dendritic arbors are similar despite altered branching hierarchies (Fig. 3g). Finally, the spine density in FLX-exposed basilar dendrites was reduced, although this differences did not reach statistical significance on spine densities across branch orders. An overall reduction in spine density was observed (Fig. 3h).

**Figure 4:**
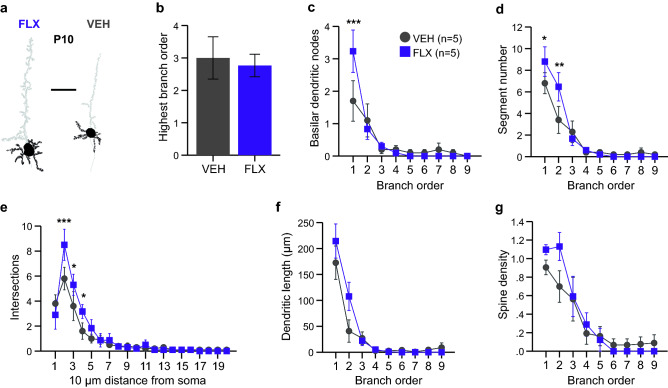
Dendritic features of mPFC layer V pyramidal neurons are increased at P10 by Maternal FLX. (**a**) Representative tracings of Golgi-Cox impregnated mPFC layer V neurons from P10 FLX and VEH brains. Areas in black indicate quantified basilar dendrites and soma. Scale bar is 50 µm. (**b**) Comparable branching orders were achieved by FLX- and VEH-exposed mPFC layer V neurons at P10 (n = 10 neurons averaged from 5 VEH mice and n = 10 neurons averaged from 5 FLX mice). (**c**) First order branching nodes were increased in FLX-exposed brains. (**d**) The number of basilar dendritic segments per branch order were increased in FLX-exposed neurons at the 1st and 2nd orders. (**e**) FLX-exposed neurons showed an increased in intersections 20–40 µm distance from the soma as assessed by Sholl analysis. (**f-g**) No difference was observed for basilar dendritic length or spine density between P10 FLX- and VEH-exposed neurons. Data are means ± SEM. Statistical significance, ***p < .001, **p < .01, *p < .05.

To determine if the abnormalities present in the adult mPFC neurons were apparent at early postnatal ages, we also examined mPFC layer V pyramidal neurons from P10 mice exposed to either FLX or VEH during neurodevelopment (Fig. 3a, 4a; Tables 2 and 3). mPFC neurons at this age were largely unaffected by FLX exposure. The soma size again was unchanged in FLX-exposed neurons (Table 3) and a similar level of branching order was achieved in both FLX- and VEH-exposed neurons (Fig. 4b. However, an increase in nodes on 1st order branches was observed in FLX-exposed neurons (Fig. 4c), which was accompanied by an increase in 1st and 2nd order branching segments (Fig. 4d). This was paralleled spatially by an increase in intersections at a 20–40 µm distance from the soma (Fig. 4e). Dendritic length was unaffected (Fig. 4f). Finally, a non-significant increase in spine density was also observed (Fig. 4g). These data suggest the impact of FLX on mPFC layer V neurons during early postnatal ages is much smaller, and in the opposite direction, than that observed in adult neurons.

## Discussion

Here we tested the hypothesis that early perturbation of the 5HT system disrupts neuronal architecture in areas involved in social behavior circuitry. Using maternal SSRI exposure as a model of early 5HT system perturbation, we observed morphological changes to layer V pyramidal neurons in areas related to social behavior deficits in the offspring. Specifically, maternal FLX resulted in less elaborate basilar dendritic branching in M1 neurons as well as reduced spine density during development, possibly due to enhanced dendritic pruning, which persisted into adulthood. We observed a similar blunting of basilar dendritic branching and spine density in mPFC neurons in adulthood, which was not present during development. Together, our morphological data indicate that 5HT perturbation during neurodevelopment through maternal FLX exposure affects dendritic spine formation and arborization in brain regions implicated in social behavior.

An important consideration for interpretation of our results is the degree of FLX transfer via placenta or lactation from the dam to the offspring CNS and whether FLX may be directly acting on the offspring 5HT system. In our previous study, we confirmed FLX reached the developing brain of the offspring via HPLC measurement of FLX and its active metabolite NFLX in whole brains of P9 pups exposed to maternal FLX^19^. We detected the presence of both FLX and NFLX in the brains of FLX-exposed pups and failed to detect either in the VEH-exposed control brains. The levels of FLX and NFLX in the pup brains were 43% and 32%, respectively, of that measured in an equal amount of dam brain tissue. These data indicate that FLX and NFLX were active in the offspring brain during maternal exposure, suggesting the developing 5HT system is directly targeted, and this activation, at least in part, is responsible for the observed phenotypes. In addition, we did not find differences in pregnancy outcomes between our dams exposed to FLX and those exposed to VEH. This further supports that the dendritic morphological changes resulted from direct action of FLX in the brains of the developing offspring and not as a result of indirect action on the health of the dam during pregnancy.

Our findings complement and extend the current work in this area. Previously, the morphology of M1 layer V pyramidal neurons were examined in rats at P35 following a short exposure duration to FLX during the first 4 days postnatal. Similar to our findings, a decrease in complexity was reported including decreased intersections in the Sholl analysis, dendritic order segments, nodes and endings, and total dendritic length^22^. However, to our knowledge, no previous work has examined persistent versus transient effects of FLX on dendrites in this area. Yet these findings at P35 together with ours at P10 and P79 in mice support the conclusion that altered 5HT activity during development due to FLX persistently disrupts the morphology of M1 layer V pyramidal neurons. In other studies of FLX exposure and morphological changes to the mPFC, results were counter to ours. Ko et al., published changes to dendritic morphology of layer II/III neurons of the mPFC with neonatal rat exposure^23^. Specifically, they report increased nodes, intersections, endings, and segment number. The contrast with our findings may stem from the difference in cortical layer or species. In addition, it is unclear from their report the age at assessment. Thus, if these rats were assessed early in development, it is plausible we observed a similar, albeit much reduced, effect in P10 mice. Previous work also highlighted other areas of the brain vulnerable to FLX-induced dendritic changes. These areas include the basolateral amygdala, the layer IV barrel cortex and other areas of the somatosensory cortex^22,24^. The sensory and social behavioral consequences of 5HT perturbation of these areas is an important future research endeavor.

Work from other groups examining the impact of early manipulations to other neurotransmitter systems on cortical neuronal morphology suggests dendritic architecture may be vulnerable to early perturbation of any system, yet the sensitivity of this vulnerability may differ. Specifically, a model of prenatal methadone exposure in which dams were injected with methadone daily from pre-gestation through offspring weaning showed unchanged morphology of M1 layered V pyramidal neurons at P21-26, as measured by Sholl analysis of intersections and length, despite altered intrinsic properties like significantly reduced firing rates and increased amplitude of excitatory responses^25^. Another model of ethanol exposure from embryonic day 0-P10 exhibited unchanged basilar intersections, larger soma area, and significantly reduced basilar spine densities of layer II/III pyramidal neurons of the mPFC at P90^26^. Finally, a model of gestational nicotine exposure showed no change in basilar dendritic branching, length, or spine density of layer III neurons of the anterior cingulate cortex or mPFC at P21, yet an increase in dendritic length in layer III neurons of the parietal cortex with no accompanying change to branching or spine density^27^.

Alterations in spine density have been observed across multiple studies of developmental FLX exposure, corroborating our findings here^22,23,28^, suggesting this may be a particularly vulnerable feature to early 5HT manipulations. Indeed, other pharmacological and genetic manipulations of the early 5HT system have also shown an influence on spines. Specifically, other drugs in the SSRI class (e.g. fluvoxamine^29^), as well as both the 5HT transporter knockout^30^ and the 5HT synthesizing enzyme tyrosine hydroxylase 2 knockout^31^ models showed differences in spine densities.

It may be that FLX’s influence on spine formation drives the social phenotypes we previously observed. In vitro work has shown that the 5HT1A and 5HT2A/C receptors directly inhibit and promote spine maturation, respectively^32^. Work from another group demonstrated that administration of a 5HT2A antagonist prevented the decrease in USV production induced by the SSRI paroxetine in rats^33^, indicating a role for this receptor in SSRI modulation of pup USVs. Taken further, it is possible that USVs are spared by the promotion of spine formation by 5HT2A activation counteracting a possible spine reduction by the SSRI. Further support for a possible connection between spine density and USVs comes from work showing that the knockdown of the human language-associated *FoxP2* target SRPX2 reduced spine density and USVs in pups^34^. Studies of the impact of learning on spine formation^35^ also indicate that such a reciprocal relationship may exist between spine density and USVs. Future studies examining spine morphology in the presence and absence of USVs are needed to directly examine the link between spine formation and USV production.

Previously, we also observed an increase in dominant displays of social hierarchy behavior in this model^19^. The mPFC exerts top-down control on 5HT release through its projections to the dorsal raphe, specifically inhibition^36^. Shifts up or down in synaptic efficacy of layer V pyramidal cells in the mPFC have been shown to increase or decrease, respectively, social dominance rank in the mouse^21^. What is more, 5HT has been linked to dominance across species, although not always in the same direction. Studies in monkeys showed that higher 5HT blood or synaptic concentrations increased dominance behavior, while depletion of 5HT resulted in more submissive behavior^37^. In addition, dominant monkeys had almost two times higher 5HT levels compared to subordinate monkeys^37^. In contrast, feeding adult mice a tryptophan-depleted diet, and thus reducing production of 5HT, resulted in increased dominance in the tube test^38^. While the connection between 5HT and dominance needs further investigation, it is possible that the altered mPFC morphology observed in our model influenced dominance by impacting mPFC control of raphe 5HT activity.

Our study adds to previous morphological characterizations of developmental 5HT exposures in multiple ways. As mentioned above, this study is the first to our knowledge to examine the effect of 5HT manipulations at an early and late age, allowing for discussion of transient versus persistent effects on dendritic morphology. Indeed, the amount of changes over time was quite marked, particularly in the mPFC. In addition, the duration of our SSRI exposure protocol encompasses the periods of gestation and lactation, covering a large period of offspring neurodevelopment. Thus, our 5HT perturbation model likely influences a broader range of neurodevelopmental processes compared to shorter exposure durations used in the previous studies^22–24,28,39–41^. Additionally, our route of drug administration was optimized so that the dam would experience little to no stress. Our oral administation of FLX through drinking water avoids the daily handling and injection stress present with intraperitoneal or subcutaneous injections^42^, and our pair-housing of the dams avoided any isolation-induced stress. Thus, we avoid the confound of maternal stress effects in the interpretation of our results. These advantages of our approach may explain why our results differ somewhat from the rat layer 2/3 results described above. A limitation of our study is the conflation of drug with litter effects, which is inherent in the design of maternal exposure studies as within-litter controls are not possible. In addition, several of our effects were too small to cross the statistical threshold of the 5% critical alpha level at the current power. For example, in adults there were differences in branch nodes and segments that echoed what had been seen in younger animals. While not significant (p < 0.056–0.08) in adults, such a marginal p-value could still be consistent with a persistent, but underpowered, phenotype. However, an alternative interpretation for these marginal effects is that the reduced basilar branching complexity is begining to resolve in adulthood. Likewise FLX exposure showed interesting adult-only trends on soma size and spine density in adult PFC that were not present in pups. For both sets of findings, additional work is require to tease apart these interpretations.

Overall, our results indicate that M1 and mPFC are vulnerable areas to changes in 5HT activity during neurodevelopment, at least at the cellular level. Whether these cellular changes underlie the behavioral deficits previously reported is unknown^19^. However, future work coupling area-specific early 5HT perturbation or GPCR activation using neuronal signal-transduction manipulations such as Designer Receptors Exclusively Activated by Designer Drugs (DREADDs)^43^ with social and sensory behavior assessments will help to further understand the level of vulnerability of these areas to perturbed signaling during development. Ultimately, this research will help us understand best practices when it comes to pharmacotherapies and the developing brain.

## Materials and methods

### Animals

All animal procedures were approved and performed in accordance with the Washington University in St. Louis Institutional Animal Care and Use Committee. The study is reported in accordance with ARRIVE guidelines ([https://arriveguidelines.org). We used C57BL/6J mice (https://www.jax.org/strain/000664; RRID: IMSR_JAX:000664) for this study. A total of 22 mice (8 females and 14 males) from 7 litters (4 FLX, 3 vehicle) were used for dendritic morphology experiments. The mice were housed in 28.5 × 17.5 × 12 cm translucent plastic cages with corn-cob bedding and standard lab diet and freely available water. The colony room lighting was a 12/12 h light/dark cycle with room temperature (~ 20–22 °C) and relative humidity (50%) automatically controlled. All mice participating in this study were bred and maintained in the vivarium at Washington University in St. Louis and were all group housed by sex after P21.

### Maternal SSRI administration

The SSRI administration protocol used here is the same as previously published^18,19^. The longer half-life of FLX, compared to other SSRIs, was ideal for this study because it allowed for safe abrupt cessation of drug exposure. Dams were exposed to FLX beginning from one week prior to mating through offspring age P14, just before pups begin to consume food and water in order to avoid direct drug exposure in the pups. FLX was delivered through drinking water sweetened with 1% saccharin at a dose equivalent to the maximum recommended human dose in mice which is 48 mg/d (16 mg/kg/d based on a 30-g mouse)^44^. The dose calculations are based on equivalent surface area dosage conversion factors^45^ and approximate drinking water consumed daily^46^. This administration route was chosen to minimize stress that can occur with daily injections. Control dams were given 1% saccharin drinking water vehicle (VEH) only. Females of the same treatment group were co-housed to reduce stress induced by isolation housing.

### Golgi-Cox impregnation and morphological analysis

The Golgi-Cox method^22^ was used to quantify dendritic morphology. This technique, while almost 150 years old, remains a standard histological technique for its ability to stain neurons in their entirety, including cell bodies, axons, dendritic processes, and synaptic spines. Importantly, Golgi-Cox stains approximately 1–2% of neurons present, resulting in clearly distinguishable neurons and processes, allowing for stereological tracing of the dendritic arbors of single neurons^47^.

#### Tissue preparation

Offspring from FLX or VEH dams were deeply anesthetized using isoflurane and then euthanized via decapitation at either P10, a developmental age at which we previously observed USV deficits, or P79, an age at which increased social dominance behaviors were observed^19^. Brains were rapidly dissected and stained via Golgi-Cox impregnation using the FD Rapid GolgiStain kit (FD NeuroTechnologies, INC, Ellicott City, MD). All procedures followed the manufacturer’s manual with minor deviations as reported below. Freshly dissected brains were rinsed with Milli-Q water and immersed in impregnation solution (equal parts A solution and B solution) mixed 24 h earlier and stored in the dark. Impregnation solution was replaced with fresh solution after the first 6 h. Incubation lasted 21 days for P10 brains and 7 days for P79 brains, with continuous agitation. Brains were transferred to Solution C for 72 h, followed by flash freezing via immersion into −70 °C isopentane, and stored at −80 °C until sectioning. Tissue was coronally sectioned at 200 µm using a Leica CM1950 cryostat and immediately mounted on gelatin-coated slides using a small drop of Solution C. Within 72 h, slide-mounted sections were stained as outlined in the manufacturer’s manual. Slides were rinsed 2 times for 4 min each in Milli-Q water, then placed in 1:1:2 mixture Solution D: Solution E: Milli-Q water for 10 min. Sections were again rinsed 2 times for 4 min each in Milli-Q water, followed by dehydration in 50%, 70% and 95% ethanol for 4 min each and then 100% ethanol 4 times for 4 min each. Sections were cleared in Citrasolv and coverslipped using Permount and allowed to dry for two weeks in the dark at 4 °C before microscopy.

#### Neuron selection, morphological quantification

For each region, age, and drug condition, at least 10 layer V pyramidal neurons were randomly selected for quantitative analysis of dendritic branching and spine densities. The selected neurons had basilar dendritic arbors that were fully impregnated with stain and were not truncated from slicing. The apical dendrite tracings were often truncated; therefore, only morphological features of the basilar dendrites were quantified. Each neuron had several spiny basilar dendrites extending from the cell body and a single apical dendrite (Figs. 1c, 2a, 3b, 4a). The selection area of the primary motor cortex (M1) was based on the location active during USV, as described by Arriaga et al.^20^ (between −0.02 mm and + 0.40 mm from bregma; Fig. 1b). At this coronal level, the anterior commissure bridges the hemispheres at + 0.13 mm^48^, therefore neurons were traced that occur in the section where the anterior commissure bridges ± one section anterior or posterior, where the limbs of the anterior commissure are well-defined but do not bridge the hemispheres. The sampled area of the medial prefrontal cortex (mPFC) (about + 1.94 mm from bregma; Fig. 3a) was motivated by studies of Wang et al.^21^, showing that the synaptic efficacy of neurons in this area mediated social dominance. This region of mPFC is posterior to the olfactory bulb, anterior to the basal ganglia^48^, and corresponded to the anterior cingulate cortex, prelimbic area and infralimbic area^21^.

All neurons were manually traced using a 60 × oil objective on a Nikon E800 Eclipse microscope and Microfire A/R camera by Optronics with a Nikon Optem 1X DC10NN adapter connected to a Dell Precision T3600 computer equipped with Neurolucida software (MBF Bioscience, Williston, VT). Based on our previously published methods, tracing involved drawing the maximum contour of the soma, following all dendrites along their entire length, and marking all visible dendritic spines^49^. Neuronal morphology was quantified according to eight measures adapted from Bianchi et al.^49^ including number of dendritic spines, total dendritic length, dendritic spine density (ratio of spines per 1 µm dendritic length), number of branching segments, number of branching nodes, number of branching segment endings, total dendritic field area (total area encompassed by the branched structure; µm^2^), soma area (µm^2^), and highest branching order. Branch order is defined by level removed from soma, such that the dendritic branches originating from the soma are considered first-order branch segments, and branches originating from first-order are second-order segments, and so on. As most apical dendrites passed out of the plane of sectioning, only basilar dendrites were included in quantitative analyses. In addition to these measures a Sholl analysis was performed, which assessed neuronal complexity as it spatially relates to the soma. In short, the Sholl analysis involved placing concentric circles with gradually increasing radii centered at the centroid of the soma. For each neuron, we tracked the number of times dendrites intersect with a circle of a given radius.

All tracings were obtained by two researchers (C.J. and S.A.), who were normed to an experienced rater (A.L.B.)^49^ and checked by the primary investigator (S.E.M.). All assessments were conducted blinded for treatment group. As assessed by the coefficient of intraclass correlation, interrater reliability was high in all measures of interest (C.J. vs. S.A.): dendritic spines = 0.817; dendritic length = 0.968; branching nodes = 0.885; branch segment endings = 0.956; and soma area = 0.805.

### Statistical analysis

All statistical analyses were performed using the IBM SPSS Statistic software (v.28). Prior to analyses, all data was screened for missing values, fit between distributions and the assumptions of univariate analysis, and homogeneity of variance. Averages were calculated for each outcome across individual neurons traced within each brain (one to four per mouse) and data analyzed at the level of mouse. Two-tailed analysis of variance (ANOVA), including one-way and repeated measures ANOVA, were used where appropriate. Pooled error variances were used when assessing simple main effects of drug across each level of repeated measures. The Huynh–Feldt adjustment was used to protect against violations of sphericity/compound symmetry assumptions where appropriate. Non-parametric testing (e.g. Mann–Whitney U) was used for comparing distributions that violated assumptions of univariate parametric analysis. Multiple pairwise comparisons were subjected to Bonferroni correction when appropriate. Critical alpha value for all analyses was *p* < 0.05. Test statistics are provided in Table 2.

## Data Availability

The datasets generated during and/or analyzed during the current study are available from the corresponding author upon reasonable request.
